# Oncolytic Bacteria and their potential role in bacterium-mediated tumour therapy: a conceptual analysis

**DOI:** 10.7150/jca.35648

**Published:** 2019-07-23

**Authors:** Yuqing Wang, Wenxuan Guo, XiaoLi Wu, Ying Zhang, Ciaran Mannion, Anatoli Brouchkov, Yan-Gao Man, Tingtao Chen

**Affiliations:** 1 Institute of Translational Medicine, Nanchang University, Nanchang, Jiangxi 330031, PR China; 2 JiangXi university of traditional Chinese medicine, College of basic medicine, Nanchang 330000, PR China; 3 Department of Molecular Microbiology and Immunology, Bloomberg School of Public Health, Johns Hopkins University, Baltimore, MD, USA; 4 Hackensack University Medical Center, Hackensack, NJ, USA; 5 Lomonosov Moscow State University, Leninskie Gory, Moscow 119991, Russia; 6 Tyumen State University, Volodarskogo 6, Tyumen 625003, Russia; 7 Department of Pathology, Hackensack Meridian Health-Hackensack University Medical Center, NJ, USA

**Keywords:** Engineered bacteria, Tumour therapy, Human microbiota, Probiotics

## Abstract

As the human microbiota has been confirmed to be of great significance in maintaining health, the dominant bacteria in them have been applied as probiotics to treat various diseases. After the detection of bacteria in tumours, which had previously been considered a sterile region, these bacteria have been isolated and genetically modified for use in tumour therapy. In this review, we sum up the main types of bacteria used in tumour therapy and reveal the mechanisms of both wild type and engineered bacteria in eliminating tumour cells, providing potential possibilities for newly detected, genetically modified, tumour-associated bacteria in anti-tumour therapy.

## 1. Introduction

The adult human body is composed of 10^13^ eukaryotic cells, while approximately ten times as many microorganisms reside on the surface and inside the body 1. Various microorganisms interact with each other, constituting the microbiota of the human body, which is essential for human health. Four dominant microbiota including gut, vagina, oral cavity and skin have been regarded taking charge of different aspects of human health 2-5. Dysbiosis of these microbiota is associated with continuous stimulation of the immune response and the production of bacterial metabolites-derived carcinogens, which increase the risk of acquiring various diseases, e.g. inflammatory bowel disease (IBD), allergy, cancer, diabetes, obesity and neurodevelopmental disorders 6-7.

Commensal microbiota exists on the surface or inside the body. Bacteria have also been detected in some regions that had previously been considered sterile, including the placenta, breast milk, tumours and blood. This suggests the potential existence of probiotics or pathogens in those areas and may provide clues for the diagnosis and treatment of tumours 8-10.

In the early 19^th^ century, wild-type *Clostridium perfringens* was first detected in patients with cancer, and live anaerobic bacteria were injected into animal models to examine their oncolytic effects 11. Then, 'smarter' bacteria (e. g. *Salmonella typhimurium*, *Listeria monocytogenes*) were designed to kill tumour cells with the development of advanced gene engineered techniques via the expression of tumour-related antigens, pro-drug-converting enzymes or agents toxic to tumours 12-14. Therefore, it is an interesting topic to reveal the microbial composition living in tumour and elucidate their potentialities as oncolytic bacteria or bacteria that secrete metabolites or proteins that are directly toxic to cancer cells 15.

## 2. Bacteria eliminating tumours — History, mechanisms and therapeutic effects

Cancer is a devastating disease defined by abnormal cell growth with the potential to invade and spread to other parts of body, which is called metastasis 16. Solid tumours account for approximately 90% of all cancers and are characterised by aberrant vascular formation 17. Formation of arteriovenous shunts and blunt ends leads to less delivery of oxygen and nutrients to overall neoplastic tissues 18. Tissues surpassing the limitation of blood delivery become hypoxic or even necrotic, which is a typical characteristic of solid tumours and a distinguishable feature between normal and neoplastic tissues 19.

All tumours contain two basic components: the parenchyma and stroma 20. The parenchyma is made up of transformed or neoplastic cells and it mainly determines the behaviour of tumour; the stroma is composed of host-derived and non-neoplastic cells, made up of connective tissue, blood vessels, and host-derived inflammatory cells 20. These two compartments of the tumour create radically different microenvironments that can profoundly influence therapeutic approaches 21. For instance, the sensitivity of hypoxic regions in the tumour to ionic radiation is one third that of normal tissues, with half the normal concentration of oxygen 22. The hypoxic region of the tumour may also be insensitive to chemical agents due to an insufficient blood supply 23. From another point of view, conventional therapies for treating cancers including radiotherapy and chemotherapy have low specificities, reflected in eliminating both normal cells and tumour cells, resulting in the suppression of the immune system 24. Thus, innovative therapies such as targeting the hypoxic area of the tumour need to be further explored for more specific and efficient cancer treatments.

The existence of bacteria in tumours provides a potential new direction for cancer therapy using wild type or engineered bacteria. The first breakthrough in cancer therapy dates back to 1813, when Vautier observed tumour regression in patients with gas gangrene after infection of *C. perfringens* 11. Several decades later, a pioneering New York surgeon, William B, Coley, dedicated himself to curing cancer patients using immunotherapy after the loss of his first cancer patient, a young girl with a sarcoma in her right arm 25. When he looked through the medical records of the hospital, he noticed a 7-year-old patient who had experienced the regression of a recurrent sarcoma after the infection of erysipelas 25. Then, he began to search the medical literature for similar situations and found the most frequent combination of infectious disease and cancer were sarcoma patients with erysipelas 25. This incidental discovery elicited continuous studies on curing cancers via the direct injection of streptococcal broth cultures of heat-killed *Streptococci*, finally reaching the achievements of Coley's toxin (heat-killed *Streptococcus* and *Serratia marcescens*) 26. In the next 40 years, he treated hundreds of patients with inoperable sarcomas using immunotherapy 27. These findings elicited interest in exploring the therapeutic effects of anaerobic bacteria existing in hypoxic regions of neoplastic tissues. Over the years, a series of obligate or facultative anaerobic bacteria have been tested, targeting tumour cells and inducing tumour regression. They may exert their anti-tumour effects via a number of mechanisms, including direct toxicity to tumour cells via type III secretions, in which cytotoxic peptides are injected directly into the target cell's cytoplasm 28,29; the facilitation of a non-specific immune response; the depletion of necessary nutrients; and the alteration of the tumour microenvironment by bacterial colonisation. Additionally, immunomodulatory effects including the stimulation of the dendritic cells or alterations in T helper cell polarisation could play a role 30,31.

**Figure 1:**
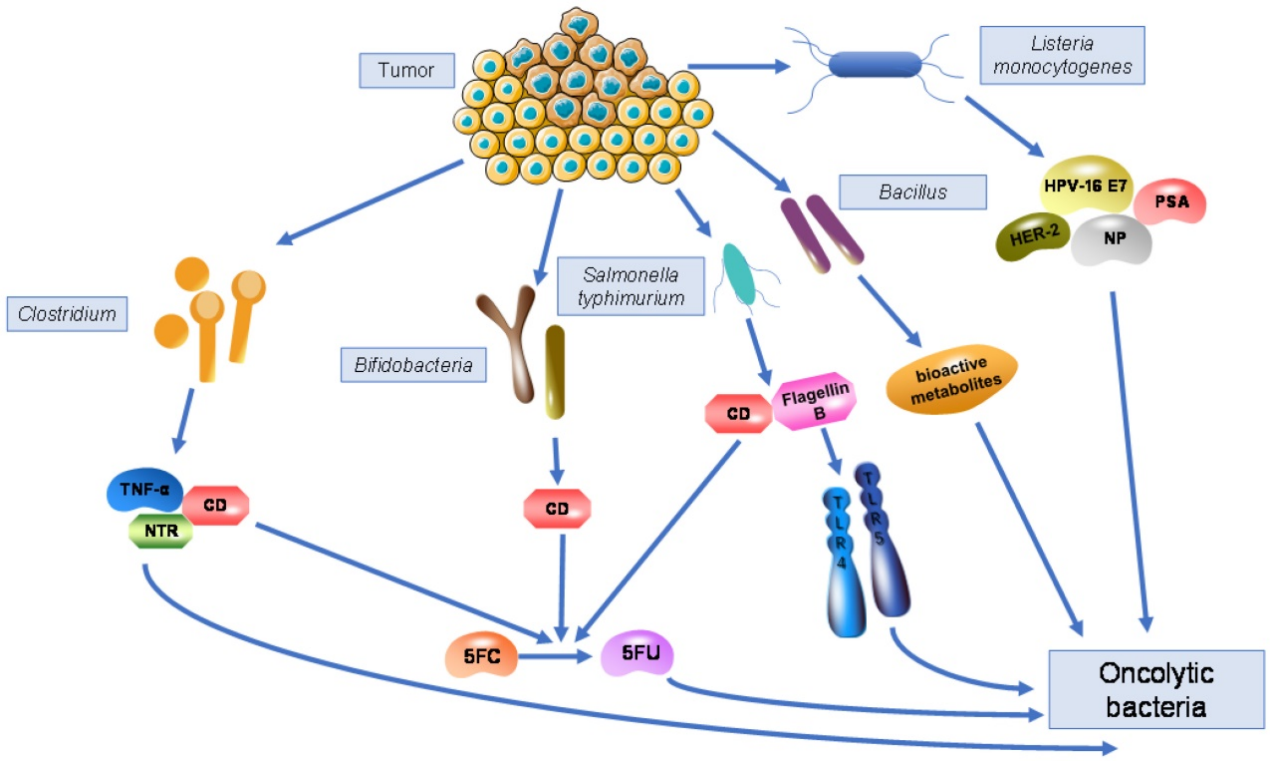
**Bacteria living in tumours and their mechanisms as oncolytic bacteria.** Genetically modified *Clostridium* are able to express cytosine deaminase (CD), which can convert the non-toxic pro-drug 5FC into the toxic antineoplastic drug 5FU, similar to *Bifidobacteria* and *Salmonella typhimurium*. Engineered *Clostridium* can also express nitroreductase (NTR) or TNF-α, killing tumour cells by converting pro-drugs or producing cytokines. Recombinant *Salmonella typhimurium* expresses flagellin B to activate TLR4 and TLR5, triggering stronger immune response to kill tumour cells. Engineered *Listeria monocytogenes* acts as a vaccine vector by expressing various antigens including NP, HPV-16 E7, PSA, and HER-2, generating cell-mediated immunity to eliminate tumours. *Bacillus* produces various bioactive metabolites against cancer cells.

The characteristics of anaerobic bacteria living in hypoxic tumour tissues allows them to exert anti-tumour effects against those cells that are resistant to other anti-cancer therapies such as radiation and chemotherapy. Bacterial motility promotes their dispersion inside the tumour and to more distant sites, which amplifies their function to some extent. Meanwhile, bacteria also have the ability carry specific genes due to their large genome size and ease of genetic manipulation. Transgenes coding for cytokines, enzymes, and immunogens can all be expressed following bacterial infection. *Clostridium* spp., *Bifidobacterium* spp.,* Salmonella typhimurium*, *Vibrio cholera*,* Listeria monocytogenes*, and *Bacillus* spp. are bacteria that have been most widely identified inside tumours and have the ability to kill tumour cells in a natural or genetically modified form, indicating their potential for therapeutic effects in anti-cancer therapy (Fig. 1).

### 2.1 *Clostridium*

*Clostridium* is a genus of obligate anaerobic, Gram-positive bacteria that has the ability to produce endospores 32. The normal, reproducing cell of *Clostridium* is called the vegetative form. Which has a rod shape. Several strains are considered to be common pathogenic bacteria, including *Clostridium botulism*, *C. difficile, C. perfringens*, and* C. tetani* 33. After Vautier found that patients with gas gangrene seemed to be cured following infection with *C. perfringens*, the toxicity and antitumour effect of many strains of *Clostridium* were tested using animal models 34. In 1927, Torrey and Kahn first reported the ability of *C. histolyticus* to lyse tumours by injecting sterile filtrates of the bacteria directly into tumours in rats, resulting in a considerable number of tumours limited to a diameter of 2 cm 35.

Later, in 1935, Connell found that the proteolytic enzymes secreted by *C. histolyticus* were able to degrade tumour cells preferentially without influencing normal tissue 36. Parker et al. in 1947 observed the apparent lysis of sarcomas in rat models after the intratumoural inoculation of spores of *C. histolyticus* 37. The precise selectivity of *Clostridia* to hypoxic/necrotic regions was further identified by Malmgren and Flanigan in 1955 38. Spores of the *C. tetani* were injected intravenously into tumour-bearing mice and non-tumour-bearing mice; the tumour-bearing mice died within 48 h after injection of the spores while the non-tumour-bearing control mice survived without any tetanus symptoms throughout the whole study 38. These experimental phenomena indicated the complete germination of spores and the production of tetanus toxins within tumour tissues, subsequently verified by microscopic examination of tumour and normal tissue sections. A non-pathogenic strain of *Clostridium* named *C. butyricum* M55 was isolated from the soil, then later renamed as *C. oncolyticum*; it is now classified as *C. sporogenes* (ATCC13732) 39. It may have the same ability to lyse tumours.

Aiming at finding an unharmful *Clostridium* oncolytic therapy, J. R. Möse and G. Möse injected spores of non-pathogenic proteolytic strain *C. butyricum* intravenously into mice transplanted with solid Ehrlich carcinomas 39. The tumours then lysed, discharging a brown liquid necrotic mass 39. However, some experimental animals still died after the extensive lysis of tumours as remaining viable cells led to the regrowth of them. Under these circumstances, in order to target the remaining cancer cells, combination therapies were created. The combination of spore administration and various chemotherapeutic agents including 5-fluorodeoxyuridine and cyclophosphamide resulted in a more obvious reduction in tumour weight 40,41. Moreover, the combination of *Clostridium* spores with local irradiation and high-frequency hyperthermia increased the level of hypoxia in the tumour and led to an evident increase in the survival rate of mice bearing melanomas 42. The effectiveness of antitumour therapy could also be achieved by decreasing the oxygen level of the air breathed by tumour-bearing mice to only 11-12%. Owing to the effectiveness of combined therapies, the tumour lysed more thoroughly, but the tumour cells could not be eliminated completely.

In 1967, J. R. Möse and G. Möse injected themselves with spore suspensions of *C. sporogenes* ATCC 13732 and verified its non-pathogenicity in humans 43. Further experiments indicated the limitations of the intravenously injecting *Clostridium* spores in five patients with neoplastic disease. Oncolysis only occurred in large tumours, not in the surrounding normal tissues or smaller tumours and metastases 43. The intratumoural injection of spores in patients with glioblastoma should be accompanied by surgery to remove the lysis abscess in the case of rupture as it may lead to death 44. *Clostridium* spore injection only affects large tumours rather than small tumours and metastases, and it cannot clear all tumour cells, which may result in the regrowth of the tumour or the discontinuation of clinical trials.

As soon as recombinant DNA techniques and specific transformation protocols for *Clostridium* became available, much effort was put into the development of *Clostridium* strains producing anti-tumour proteins. Because of their exquisite specificity to germinate in the hypoxic/necrotic regions of solid tumours, these strains could be used as vector systems for the specific delivery of therapeutic proteins in the tumour microenvironment 45. In preclinical studies, Schlechte and Elbe were the first to attempt the combination of the *C. sporogenes* ATCC 13732 with the *Escherichia coli*-derived gene coding colicin E3, a bacteriocin with cancerostatic properties 46. Later, genetically modified *C. beijerinckii* was capable of expressing *E. coli* enzymes nitroreductase and cytosine deaminase, which converts the non-toxic pro-drugs CB1954 and 5-fluorocytosine into toxic anti-cancer compounds, which diffuse into the tumour and trigger cell death by interfering in DNA replication/transcription 45,47. In 2001, the same principle of producing cytotoxic agents was applied to recombinant *Clostridium* with the promotor recA driving the expression of the gene encoding for the cytokine TNF-α after 2 Gy of irradiation, indicating a promising prospect for gene targeting and ionising radiation 48. To select the anaerobic bacterial strain with the highest efficacy in growing within avascular tumours, a systemic assessment was done among the 26 strains, and *C. novyi* was the most promising 42. The spores of engineered *C. novyi* without its lethal toxin (*C. novyi-NT*) were intravenously injected with chemotherapeutic drugs, resulting in extensive necrosis of transplanted tumours within 24 hours 49. DNA damaging agents (Cytoxan, mitomycin) as well as tumour vascular collapsing agents (Dolastatin-10) were combined with *C. novyi-NT* and led to the dramatic regression of transplanted tumours in mice 49. This strategy was termed combination bacteriolytic therapy (COBLT) and may lead to an innovative direction for cancer therapy by i,v. injection of *C. novyi-NT* recombinated with specific anti-tumour genes.

### 2.2 *Bifidobacteria*

*Bifidobacterium* is a genus of Gram-positive, obligate anaerobic bacteria. They are naturally present in the dominant colonic microbiota, and represent up to 25% of the cultivable faecal bacteria in adults and 80% in infants 50. It is regarded as potentially health-enhancing bacteria in the human gut and its dominance in the faeces of breast-fed babies is considered to impart protection against infections 51. As a probiotic agent, *Bifidobacterium* is present in fermented foodstuffs (e.g., yogurt, cheese, olives and other fermented vegetables) and its safety is supported by those foodstuffs and growing knowledge about *Bifidobacteria* taxonomy and physiology 52.

*Bifidobacterium* is considered as a safe candidate carrier due to the bacterial medicine *Bifidobacterium bifidum* (LacB; Nikken Kagaku, Tokyo, Japan), which is prescribed for patients in Japan. Experiments showed that after the intravenous administration of a Lac B suspension in tumour-bearing mice, they did not show any adverse symptoms 53. The bacteria completely disappeared in non-malignant tissues such as the liver, kidney, spleen, lung, blood and bone marrow within in 24 to 96 hours and the bacteria only grow in tumour tissues 54. Later, a more advanced tumour-targeting plasmid vector (pBLES100-S-eCD) was accomplished by the combination of a promoter from a gene coding histone-like protein and the cytosine deaminase gene of *E. coli* (e-CD) 55. The function of this gene is to concert 5-fluorocytosin (5FC), an antifungal reagent of low toxicity, into 5-fluorouracil (5FU), a common anticancer drug specifically for targeting tumour tissues 55. In early cancer treatment experiments, autochthonous tumours of rat breast cancer were developed with the carcinogen 7,12-dimethylbenz(a)anthracene 56. The suppression of tumour growth was observed in the group treated with the injection of bacteria transformed by e-CD-expression vectors, and when the pro-drug 5FC was given orally 56. The same treatment system was also found to be efficient on human breast cancers transplanted into immunologically-deficient nude mice 57.

In order to examine the immunological reactions, further tests concentrated on inflammatory cytokine levels in the blood after the i.v. injection of *B. longum* carrying the e-CD expression vector or non-pathogenic *E. coli* as a control. The results showed that no inflammatory cytokines were induced by the injection of *B. longum* while* E. coli* induced the cytokines such as the interleukin-6 (IL-6), IL-1β and IL-18. This experiment demonstrated that the genetically modified *B. longum* did not trigger intense immunological response, thus verifying its safety. Similar results have also been reported showing that *Bifidobacteria* induces low levels of IL-12 and TNF-α in the blood regardless of the strain used. In contrast, strains *Lactobacillus* possess different abilities to induce IL-12 and TNF-α 58.

### 2.3 *Salmonella typhimurium*

*Salmonella typhimurium* is a pathogenic, Gram-negative, facultative anaerobic bacterial species that can be found predominantly in the intestinal lumen. Its toxicity is due to an outer membrane consisting of abundant lipopolysaccharides (LPS) which protect the bacteria from the environment. The LPS is made up of an O-antigen, a polysaccharide core and lipid A, which connects it to the outer membrane 59. Lipid A is made up of two phosphorylated glucosamines which are attached to fatty acids. These phosphate groups determine bacterial toxicity. Animals carry an enzyme that specifically removes these phosphate groups to protect themselves from these pathogens. The O-antigen, being on the outermost part of the LPS complex is responsible for the host immune response 60. *S. typhimurium* has the ability to undergo acetylation of this O-antigen, which changes its conformation and makes it difficult for antibodies to recognise 61.

Wild-type Salmonella, in particular *S. typhimurium*, have been detected as the pathogen that causes self-limited enteritis in most healthy adults, infects many mammalian species and can easily be manipulated to carry therapeutic transgenes 62. *S. typhimurium* exists as a facultative anaerobe, allowing it to survive in both oxygenated and hypoxic conditions; thus, it may colonise both small metastatic lesions and larger tumours. In 1997, Pawelek et al. found that *Salmonella* has the ability to infect and accumulate within implanted tumours in mice, achieving the ratios of concentrations in tumour/normal tissue up to 1,000:1, which indicates that this may be a clinically useful anti-cancer agent 63. However, the toxicity of wild-type *S. typhimurium* limits its potential in preclinical studies of tumour therapy. For the earliest detection of attenuated *Salmonella*, Bacons and his co-workers reported the *Salmonella* with auxotrophic mutants had a great reduction in virulence 64,65. To develop a clinical candidate with higher safety, a wild-type *S. typhimurium* has been attenuated by partial deletion of the *msbB* gene, which is responsible for addition of a terminal myristyl group to lipid A 66. This mutant leads to a diminished ability of *S. typhimurium* to induce the secretion of TNF-α *in vitro* in human monocytes and* in vivo* after the administration to mice and pigs 67. For further safety consideration, the bacteria were further attenuated by partial deletion of the *purI* gene, creating a growth requirement for external sources of purines, whose concentrations are high in the interstitial tissues in the tumour environment with the attenuated toxicities identified in mutant strains 68. Genetically modified *S. typhimurium* (VNP20009) has a safety profile of possessing partial deletion of both *msbB* and *purI* genes, which limits the toxicity of *S. typhimurium* in normal tissues while retaining the tumour-targeting and tumour-inhibiting properties 68-70.

In preclinical experiments, the ability of attenuated *Salmonella spp.* to retard tumour growth was assessed in various animal models. When single-cell suspensions of *Salmonella* were intravenously injected into animals with 4-5 mm tumour or in the case of metastatic models, the growth of the tumour and dissemination of metastases were strongly inhibited for a prolonged period 71. Several models showed a longer survival time; however, the tumour eventually recurred, leading to the death of the animals 71. Site-related and systemic toxicities reported in preclinical studies of VNP20009 were not observed unless the doses of suspensions were excessive 72. Meanwhile, the specificity of VNP20009 accumulating preferentially inside tumours over the liver was also observed in different types of tumour-bearing mice at a ratio ≥1000:1. However, in normal mice and cynomolgus monkeys, VNP20009 is rapidly cleared from a peak level to undetectable in blood within 24 h, which indicates little potential for septic shock.

The mechanisms that lead to the accumulation of bacteria in tumours compared to relative lower levels in other tissues may include the different vascular structures and blood flow patterns, which may lead to the entrapment of bacteria in tumours. Additionally, bacteria may also attach or be phagocytosed by immune cells transferred to tumour sites 73.

Concerning the clinical trials about attenuated *Salmonella*, recent studies show better therapeutic effects in combination with other anti-tumour therapies. As the bacteria alone could only delay the tumour growth rather than inhibit the recurrence of tumours, more rigorous genetic modulations could be applied to *S. typhimurium* to enhance the tumour inflammatory response. These strategies include the delivery of genes coding for cytokines, pro-drug-converting enzymes and agents toxic to tumour. For instance, engineered attenuated *S. typhimurium* is able to express cytosine deaminase, which could possibly catalyse the non-toxic pro-drug 5-fluorocytosine to anti-tumour drug fluorouracil, which mimics uracil in tumour cell replication and inhibit this process 13. Another mechanism that is worth paying attention to is the activation of immune response induced by intravenously injection of genetically recombinant *S. typhimurium* detected in mice models. In this situation, an attenuated *S. typhimurium* was engineered to express a Toll-like receptor (TLR) 5 agonist* Vibrio vulnificus* flagellin B protein (FlaB), leading to the recognition of lipopolysaccharide on the surface of the *S. typhimurium* and triggering an immune response inside the tumour; the efficacy was stronger than with a single TLR4 or TLR5 agonist 74. In this experiments, scientisits found that TLR4 signalling pathway is prerequisite in triggering the anti-tumour immune response and FlaB/TLR5 pathway augment this reaction 74. This process is achieved by initial colonization of the bacteria which recruits abuntdant immune cells including macrophages and neutrophils via TLR4 signalling pathway and further activation of these immune cells triggered by TLR5 signalling mediated by the secretion of FlaB 74, Other engineered *S. typhimurium* expressing the cytokine LIGHT was injected intravenously to mice models of both primary and established pulmonary metastases, carrying out anti-tumour effects by binding to LIGHT receptors, transducing signals to induce the expression of chemokines 75. Various chemokines trigger the emigration of dendritic cells, natural killer cells, T and B lymphocytes, leading to the obvious reduced growth of primary tumours and inhibition of tumour metastases 35, 75, Genes that express haemolysin E recombinated with a highly hypoxia-inducible promoter increased tumour necrosis and reduced tumour growth by i.v. injection to 4T1 mice models 76.

The genes that can be chosen should maintain a high tumour to normal tissue ratio, resulting in the largest beneficial effect to patients. Meanwhile, genes delivered to the bacteria should have very low toxicity or even no influence on normal tissues. Ideally, the secreted substance or the activated drug should have a short circulating half-life and exert anti-tumour effects. It should be also shown that the synergetic toxic substances produced by bacteria do not influence the replication and persistence of the bacteria in tumours. It may happen that toxicities of expressing substances evolve from the modification of enzymes inside our bodies. If it comes to the situation that toxicities are too strong to sustain, it may be possible for patients to kill the bacteria and eliminate the toxicity using antibiotics. Even under the worst circumstances, the tumour would be exposed to far greater concentrations of the agent than normal tissue and presumably even limited exposure might have a substantial therapeutic benefit.

With the numerous effector genes that could be engineered into bacterial hosts, therapies could be extended to sequential or concurrent administration of similar or different bacteria that contain separate gene products. Demonstration of the central concept of selective intratumoural accumulation of bacteria in cancer patients can be expected to lead to a vast and novel repertoire of therapeutic options for the treatment of metastatic diseases.

### 2.4 *Listeria monocytogenes*

*Listeria monocytogenes* is a Gram-positive, facultative anaerobic, rod-shaped bacterium that has tumbling motility and is unable to form spores 77.

As a facultative anaerobic intracellular bacterium, *L. monocytogenes* lives and replicates within the cytosol of the host cell. It is one of the most virulent foodborne pathogens and is usually phagocytosed by antigen-presenting cells (APC). Once phagocytosed, the vast majority of the bacteria will be degraded inside the phagosome; however, about 10% of the bacteria are able to take advantage of virulent factors to break down the phagosome, which enables them to live freely in the host cytosol. These virulence factors mainly include liseriolysin O (LLO), which is a pore-forming haemolysin and phospholipase 75. Once the bacterium enters the cytosol, actin polymerase A (Act A) is synthesised and *L. monocytogenes* starts to undergo rapid replication 78. Act A is responsible for the recruitment and polymerisation of host cell actin, leading to the formation of a tail of actin, allowing for intracellular and intercellular movements 79. Reorganisation of the cytoskeleton of host cells produces pseudopods containing *L. monocytogenes* that extend towards nearby cells, forming a double-membrane protrusions and infecting them 80. Through this mechanism of spreading to other cells, *L. monocytogenes* is not exposed to the extracellular defences of the host, primarily accounting for why host cells do not develop a protective humoral response to it. The genes related to this cycle (i.e., *prfA*, *plcA*, *hly*, *mpl*, *actA*, *plcB*, *inlA*, *inlB*, *inlC* and *hpt*) are regulated by the transcriptional activator listeriolysin A (PrfA) 80. It is possible that these virulence factors could enhance the immunogenicity of tumour-associated antigens, which are poorly immunogenic.

Considering the host immune response to *L. monocytogenes*, antibodies have been regarded for a long time to play no role in *Listeria* resistance, as there have been studies showing no protective effects after transferring the serum of *Listeria*-immune mice to naïve mice 81,82. However, Unanue and colleagues have demonstrated a mechanism of intracellular neutralisation of the secreted virulence factor LLO via anti-LLO antibodies, taken up by macrophages through endocytosis, stored in endosomal compartments, waiting for an encounter with vacuole-containing *Listeria* 83. Under these circumstances, the rate of *L. monocytogenes* escape from the phagosome is considerably reduced due to the opsonisation of LLO, thereby limiting intracellular growth. However, the amount of anti-LLO antibody used by Unanue and colleagues in these studies far exceeded physiological levels, putting into question to what extent the antibody can work well in neutralising the bacteria and demonstrating the humoral response may be too weak to provide effective protection to *Listeria* infection 83,84. Although about 10% of *L. monocytogenes* can spread to nearby hosts, the rest of the bacteria are killed through a series of mechanisms mediated by our immune systems. Early resistance to *L. monocytogenes* is controlled mainly by macrophages, neutrophils and NK cells through various cytokines and chemokines 85. Macrophages and neutrophils secrete IL-1, IL-6, IL-12, TNF-α and chemokines, recruiting natural killer (NK) cells that produce the macrophage-activating cytokine interferon-γ (IFN-γ); macrophages are responsible for further elimination of the pathogen 85. After the innate immune response, most *L. monocytogenes* phagocytised by macrophages are degraded after fusion of the phagosome and lysosome and protein fragments are presented on major histocompatibility complex (MHC) II molecules induced by IFN-γ, which in turn present antigen to CD4^+^ T cells. The remaining 10% of *L. monocytogenes* escape from the phagolysosome and replicate inside the cytosol. The proteins produced here are degraded inside the proteasome and transported into the lumen of the endoplasmic reticulum (ER) through the transporter associated with antigen processing (TAP) and loaded onto MHC I molecules, thereby triggering the CD8^+^ T cell response 1. Thus, *L. monocytogenes* is an ideal vector for delivering antigens to be processed and presented through both the MHC class I and II antigen processing pathways, which is a hallmark of host immunity to *L. monocytogenes*.

Owing to this property, unusual among intracellular pathogens, *L. monocytogenes* has been used as a vector to generate cell-mediated immunity against a wide range of pathogen antigens: influenza nucleoprotein (NP), HPV E7, HIV gag, simian immunodeficiency virus (SIV) env, tumour antigens tyrosinase-related protein (trp)-2 and human epidermal growth factor receptor (HER)-2/neu in preclinical experiments by normally i.v. injection. As a vaccine vector, the recombinant *L. monocytogenes* strains that have been developed for clinical uses are highly attenuated compared to wild type *Listeria.* With attenuation, the existing anti-vector immunity does not affect the therapeutic efficacy, which was verified by using a mutant *Listeria* missing the *actA* gene as the antigen vector 78.

For the purposes of generating a CD8^+^ T cell response that is strong enough to kill tumour cells, the antigen that is responsible for triggering the immune response needs to be secreted outside the cell wall of the bacteria. In order to achieve this goal, *L. monocytogenes* is always engineered in a way by fusing target antigens with a secreted bacterial protein such as LLO to form a fusion protein. The promoter and signal sequence of LLO have been used to increase the expression of the target antigen 87-89. In addition, the promoter and signal sequences of the secreted protein (LLO and ActA) have also been applied to enhance the expression of antigens integrated into chromosomes 90. The expression of fusion proteins can be achieved using plasmids or recombined chromosomes. However, in a mouse model of HPV-derived cancer, the capacity of producing antigens using plasmid surpassed that chromosomes 88.

The first test of *L. monocytogenes* as a vector for cancer immunotherapy was carried out in a recombinant influenza NP (Lm-NP) against several tumour cell lines (CT26, Renca, B16-F10) 86,88,89. Lm-NP protected the further challenge from tumours and induced complete regression of tumours expressing the same antigen. In addition, the anti-tumour effects of Lm-NP were analysed based on the immune response, showing the mutual participation of both CD4^+^ and CD8^+^ T cells, demonstrating a potent anti-tumour immunity triggered by recombinant *L. monocytogenes* 89,91,92.

Based on this successful approach, *L. monocytogenes* was utilised to carry tumour-associated antigens HPV-16 E7, which can be either part of the *Listeria* genome following the haemolysin signal sequence (Lm-E7) or recombined into the plasmid with *hly* promotor followed by a non-haemolytic fusion of LLO-E7 (Lm-LLO-E7). Both of the strains were tested in established TC-1 tumours, derived from murine lung epithelial cells immortalised by HPV-16 E6 and E7 as well as transformed with activated oncogene *ras* 93. Results showed that Lm-LLO-E7, rather than LLO-E7, induced the regression of E7-expressing tumours, indicating their significant differences between inducing immune response.

Despite the preclinical studies carried out in animal models, engineered *L. monocytogenes* are designed to express tumour-related antigens to trigger both innate and adaptive immunity clinically 94. Attenuated *L. monocytogenes* vaccines expressing human papilloma virus (HPV) serotype 16 E7 (ADXS11-001), which is known to be overexpressed in HPV-related cervical cancer, have reached phase III clinical trials. Additionally, live-attenuated strains (ADXS31-142) that express prostatic specific antigen (PSA) and HER-2 expressing vaccines are in phase I/II clinical trials. Attenuated *L. monocytogenes* vaccines expressing mesothelin (ANZ-100/CRS-207) is being tested in mesothelioma, pancreatic, ovarian and Fallopian cancers 12.

### 2.5 *Bacillus*

*Bacillus spp.* is a genus of Gram-positive, rod-shaped, obligate aerobic or facultative anaerobic bacteria that can form spores 95. This genus of bacteria is ubiquitous in nature and has the highest bioactivity in producing hundreds of metabolites 96. These metabolites, including polypeptides, lipopeptides, polyketides, fatty acids and isocoumarin derived from *Bacillus spp.* are relatively abundant, exhibiting antifungal, anti-bacterial and even anti-tumour activities 97,98.

*Bacillus thuringiensis*, isolated firstly as a pathogen in the sotto disease of the silkworm, *Bombyx mori*, has the ability to produce crystalline parasporal inclusions when sporulating 99. The inclusions were found in the 20^th^ century to possess strong insecticidal effects to several insects 100. Proteins inside the inclusions include δ-endotoxins containing two families of insecticidal molecules, Cry and Cyt proteins 101. Cry proteins are toxic specifically to insects, while Cyt proteins have wider cytolytic effects on vertebrate and invertebrate cells, including insect cells and mammalian erythrocytes 101.

Although *B. thuringiensis* has garnered the interest of scientists as an insecticide for decades, while recent studies reveal that the most common *B. thuringiensis* strains distributed in nature are non-insecticidal, raising the question as to whether the parasporal inclusions of non-insecticidal *B. thuringiensis* have specific bioactivities 102-104. In this situation, a unique protein that is competent at targeting and killing cancer cells was identified.

In 1999, Mizuki et al. first screened the proteins inside the parasporal inclusions of 1744 strains of *B. thuringiensis*, containing 1700 Japanese isolates from Kyushu University and 44 reference types from the Institut Pasteur, Paris 105. After a series of experiments including cytotoxicity assays, hemolysis assays, and insecticidal activity tests, Mizuki et al. selected and purified three Japanese isolates (84-HS-1-11, 89-T-26-17 and 90-F-45-14) that were neither insecticidal nor haemolytic, choosing for further exploration of their cancer cell toxicities 105. After experiments on cancer-cell toxicities carried out in MOLT-4 (human leukaemia T cells), A549 (human lung cancer cells and HeLa (human uterine cervix cancer cells), all these strains exhibited strong cytocidal effects with variable toxic spectra and cytotoxicity 104. Significantly, this experiment showed that the proteins of 84-HS-1-11 and 89-T-26-17 killed MOLT-4 cells more readily that normal T cells, suggesting the specificities of these proteins 105. A year later, Mizuki et al. further obtained and characterised the protein from the inclusions of 84-HS-1-11, defining it as a toxin against human cancer cells, creating a new family of δ-endotoxins of *B. thuringiensis*, named parasporins (PS) 106. These are polypeptides with a predicted molecular weight of about 81 kDa, exhibiting cytocidal activities only when digested by proteases 106. According to the Committee of Parasporin in Classification and Nomenclature (http://parasporin.fitc.pref.fukuoka.jp/), the 81 kDa parasporin characterised by* Mizuki* et al. in 2000 constitutes the PS1Aa1 family. Currently, this protein group is subdivided into six major families (PS1-PS6) 107. These six families of proteins possess markedly different mechanisms against cancer cells and are activated by diverse terminal digestions 107. Concerning the primitively identified parasprorin PS1Aa1, it has been confirmed to be toxic to HeLa, MOLT-4, HL-60 (promyelocytic leukaemia cell) and HepG2 (hepatocellular carcinoma) cells 108. Further investigations revealed the possible mechanisms of PS1Aa1 in inducing the death of cancer cells. One of the most striking aspects was the early and rapid increase in the concentration of intracellular Ca^2+^, with no change in plasma membrane permeability, leading to the apoptosis of cancer cells 109. Although the anti-cancer effects of *B. thuringiensis* have only been tested *in vitro*, they pave the ways for further investigations in treating cancer *in vivo*.

Beyond *B. thuringiensis*, another closely related strain is *B. cereus*. The only discrimination between them is the crystalline proteinaceous inclusions produced by *B. thuringiensis* 110. It was originally regarded as a pathogen causing mild food poisoning due to the production of enterotoxins and emetic toxins; however, not all strains carry these genes 111. Some strains of *B. cereus* have been identified as probiotics when administered in certain amounts orally 112. Probiotics act as bioactive bacteria via metabolites that are capable of exhibiting anti-bacterial, anti-viral and anti-cancer effects 113. Later, anti-cancer metabolites were obtained from an Indian *B. cereus*; fraction BC1 showed cytotoxicity against HepG2 cells by damaging DNA and triggering apoptosis in liver cancer cells 114. However, in contrast to *B. thuringiensis*, this fraction did not contain proteins 114.

Other species of *Bacillus*, including *B. subtilis*, *B. licheniformis* and so on have also been discovered to induce cytotoxicity in cancer cells via various compounds 115-117. All these observations indicate that, as a member of the phylum Firmicutes, *Bacillus* possesses strong bioactivity by producing various compounds that are toxic to cancer cells *in vitro*, providing direction for further investigations into the metabolites of *Bacillus* in the treatment of cancer.

## 3. Tumour associated bacteria and their potential as oncolytic bacteria

*Clostridium* spp., *Bifidobacterium* spp.,* S. typhimurium*, *Vibrio cholera*,* E. coli* and* L. monocytogenes* have been observed to live in tumours; some of them have been applied to treat tumours using wild type or gene-modified strains 11,118-120. *S. pyogenes OK-432*, a poorly virulent strain of type III group A *S. pyogenes* that can be treated with penicillin G, has been used as agent to treat unresectable lymphangiomas. *Mycobacterium bovis* (BCG), acquired from a virulent strain of *M. bovis* by incubating it on a special medium, can be used to treat bladder cancer 121,122. However, these two strains are mostly used after resection, acting as a complementary therapy to prevent recurrence.

Although existing oncolytic bacteria are under perpetual development, various challenges remain before they can be used successfully in the clinic. Problems include the concentration of toxic agents, targeting efficacy, bacterial toxicity, genetic instability and the combination with other anti-cancer therapies. Considering the concentration of toxic agents produced by bacteria in tumours, it should be high enough to exert therapeutic effects but not induce systemic toxicity. Targeting efficacy is influenced by tumour size, location and blood supply, which is distinct in different patients 34. In metastatic tumours, bacteria can be amenable only when hypoxic regions exist. Although bacterial toxicities have been tested in animal models and human trials, the clinical safety is not assured due to the compromised immunity of cancer patients 43,72,123. Another major problem is genetic instability, which may lead to the loss of functionalities including ineffectiveness or harmful phenotypes 124. Meanwhile, determining the proper combination of bacteria with other traditional anti-cancer therapies will be vital for eliminating all tumour cells, including metastases 49,125,126. Besides these problems, due to the distinct pathophysiology of different tumours, the selection of the most effective oncolytic bacteria against a certain type of tumour remains to be worked out. Oncolytic bacteria that have been tested in pre-clinical or clinical studies do not possess tumour specificities, which may be a hurdle for mass propagation in tumours. Therefore, eliminating the barriers of detected oncolytic bacteria and finding new oncolytic bacteria that possess tumour specificities are significant challenges.

Upon discovering oncolytic bacteria living in tumours, scientists found that some bacteria show specificities for certain types of tumours. *F. nucleatum*, which is indigenous to the oral cavity, has also been found in the gut microbiota and shows associations with colorectal carcinomas. Comparing the microbiome of colorectal carcinoma with that of a healthy colon, high enrichment of *F. nucleatum* in colonic tissues as well as stools of patients with colorectal adenomas and adenocarcinomas has been identified by metagenomic analyses 127. Studies focusing on the mechanisms of *F. nucleatum*-associated colorectal cancers have revealed that the influence of tumourigenesis is not exerted by inducing inflammation or exacerbating colitis-associated colorectal cancer; rather, it is conducted by increasing the infiltration of CD11b^+^ myeloid cells, a phenotype of myeloid-derived suppressor cells (MDSCs) in mice, which facilitate tumour growth and angiogenesis 127,128. Meanwhile, studies have suggested that an increase of specific subsets of MDSCs accounts for effective potency by suppressing CD4^+^ T cells through the expression of arginase 1 and inducible nitric oxide synthase (iNOS) 127,129. Previous studies have supported the idea that the tumour-associated neutrophils (TAN) promote tumour progression and metastasis by secreting elastase, thereby hydrolysing insulin receptor substrate 1 (IRS1), which blocks the interaction between phosphoinositide 3-kinase (PI3K) and platelet-derived growth factor receptor (PDGFR), thus activating the PDGFR-PI3K pathway 130. Similarly, tumour-associated macrophages (TAM) have been verified as potent drivers of tumour angiogenesis 131. The number of TAN and TAM increase significantly in *F. nucleatum*-fed mice by a mean of 13.4 times and 7.8 times, respectively, compared with controls, which indicates the modulatory effects of *F. nucleatum* to tumour-immune microenvironments, thereby promoting tumour progression and metastasis 127,132.

After collecting supportive data from the mouse models, the immune cell biomarker genes related to *F. nucleatum*-associated colorectal cancers in human were identified. They include TAM-associated genes (IL-6, IL-8, CXCL10), MDSC-associated genes (CD33, IL-6), PTGS2 (COX2), IL-1β and TNF-α. Their expression levels correlate with the abundance of *F. nucleatum* in *Fusobacterium*-associated tumours and some of these genes suggest an NFκB-driven proinflammatory response (PTGS-2, IL-6, IL-8, IL-1β, TNF-α). This signalling pathway was later found to be more highly activated with a higher abundance of *F. nucleatum* in tumours 127.

In another similar situation, Straussman and colleagues detected the existence of bacteria in samples of human pancreatic ductal adenocarcinomas (PDACs) and showed that the most common species, accounting for more than half of all reads, belong to the Gammaproteobacterium, in which members of *Enterobacteriaceae* and *Pseudomonadaceae* are dominant 133. Meanwhile, the potency of these bacteria in producing cytidine deaminase, an enzyme that has the ability to deaminate the chemotherapeutic drug gemcitabine, which mediates drug resistance, was confirmed in this study 133.

Based on the extensive tumour-specific colonisation of these bacteria, together with the rapid development of gene-modification techniques, bacteria living in certain types of tumour such as *F. nucleatum* and Gammaproteobacteria have excellent potential to be remoulded directionally for anti-tumour therapy. With the mechanisms of these bacteria in promoting tumour progression being revealed, the genes responsible for these processes can be selectively knocked out and recombined with genes expressing tumour-associated antigens, enzymes converting pro-drugs or agents toxic to tumours. By taking advantage of their characteristics of targeting and colonising certain types of tumours, incorporated with genetic modifications, bacteria living inside specific tumours have excellent potential for treating cancer and represent an innovative direction in complementary and direct anti-tumour therapy.
